# Are Non-Six-Membered Ring Defects Formed in Single-Walled Carbon Nanotubes Treated by a Fluorination–Defluorination Process?

**DOI:** 10.3390/nano13061086

**Published:** 2023-03-17

**Authors:** Yoji Omoto, Hiromu Morita, Yoshinori Sato, Tetsuo Nishida, Kenichi Motomiya, Hirokazu Katsui, Takashi Goto, Yoshinori Sato

**Affiliations:** 1 Graduate School of Environmental Studies, Tohoku University, Aoba 6-6-20, Aramaki, Aoba-ku, Sendai 980-8579, Japan; 2 STELLA CHEMIFA CORPORATION, 7-227, Kaisan-cho, Sakai-ku, Sakai 595-0982, Japan; 3 Institute for Materials Research, Tohoku University, 2-1-1 Katahira, Aoba-ku, Sendai 980-8577, Japan; 4 Extreme Energy-Density Research Institute, Nagaoka University of Technology, 1603-1 Kamitomioka, Nagaoka 940-2188, Japan; 5 New Industry Creation Hatchery Center (NICHe), Tohoku University, Aoba 6–6-10, Aramaki, Aoba-ku, Sendai 980-8579, Japan

**Keywords:** single-walled carbon nanotubes, non-six-membered ring defects, fluorination, defluorination, water adsorption, oxygen adsorption, conductivity

## Abstract

Single-walled carbon nanotubes (SWCNTs) modified by introducing non-six-membered ring defects, such as five- and seven-membered rings, have attracted considerable attention because their conductivity is enhanced by increasing the electronic density of states at the Fermi energy level. However, no preparation method exists to efficiently introduce non-six-membered ring defects into SWCNTs. Herein, we attempt to introduce non-six-membered ring defects into SWCNTs by defect rearrangement of the nanotube framework using a fluorination–defluorination process. Defect-introduced SWCNTs were fabricated from SWCNTs fluorinated at 25 °C for different reaction times. Their structures were evaluated, and their conductivities were measured by operating a temperature program. Structural analysis of the defect-induced SWCNTs using X-ray photoelectron spectroscopy, Raman spectroscopy, high-resolution transmission electron microscopy, and visible–near-infrared spectroscopy did not reveal the presence of non-six-membered ring defects in the SWCNTs but indicated the introduction of vacancy defects. Meanwhile, conductivity measurements performed by operating a temperature program showed that the defluorinated SWCNTs prepared from SWCNTs fluorinated for 3 min (deF-RT-3m) exhibited decreased conductivity owing to the adsorption of water molecules to non-six-membered ring defects, thereby implying the possibility of non-six-membered ring defects being introduced into deF-RT-3m.

## 1. Introduction

### 1.1. Single-Walled Carbon Nanotubes

Single-walled carbon nanotubes (SWCNTs) are one-dimensional cylindrical structures made of graphene sheets. The length and diameter of SWCNTs are several micrometers and a few nanometers, respectively, and their aspect ratio and specific surface area are extremely high. Since the initial reports of their synthesis [1,2], SWCNTs have attracted considerable attention as a fundamental material in nanotechnology. SWCNTs possess unique electrical conductivity, mechanical properties, and high current density owing to their one-dimensional structure. In particular, their electrical properties are known to be metallic or semiconducting, depending on how the graphene sheet is wound around the tube axis (chirality) [3,4,5,6,7,8]. Therefore, these excellent properties make SWCNTs promising for applications in electronic devices, such as nanowires [9], components in field-effect transistors [10], gas sensors [11,12], transparent electrodes [13], and field-emission electron sources [14].

SWCNTs are the ultimate surface materials because their electronic density of states (DOS) can be changed by simply changing their chirality. Thus, the presence of defects in SWCNTs strongly affects their electronic properties [15]. Although CNTs have been considered as nanomaterials with a perfect crystalline structure, various defects, such as topological, rehybridization, and vacancy defects (incomplete bonding defects), reportedly exist in their carbon framework [16]. 

### 1.2. Vacancy Defects

A structure lacking carbon atoms in the framework is called a vacancy defect. Vacancy defects are inherent to the synthesis of nanotubes. In particular, SWCNTs synthesized by chemical vapor deposition (CVD) have numerous vacancy defects. Although vacancy defects are also present in SWCNTs synthesized by the arc discharge method, the number of vacancy defects is much smaller than that in SWCNTs synthesized by CVD. Vacancy defects are also introduced during the purification of SWCNTs by acid treatment or combustion in air [17]. 

Vacancy defects exist not only at the several-atom level but also in large defects with tens of atoms missing. Acid treatment with sulfuric or nitric acid introduces large vacancy defects into nanotubes owing to the partial oxidative etching of carbon atoms [18]. When materials are irradiated with electron beams that have kinetic energies exceeding a certain threshold value, a phenomenon called “knock-on damage” occurs, in which atoms in the materials are physically repelled. For SWCNTs, the skeletal carbon atoms are ejected by incident electrons with a kinetic energy higher than 86 keV, and vacancy defects are induced [19]. Therefore, transmission electron microscopy (TEM) has been performed using electron beams below 86 keV, which are considered to cause no damage to SWCNTs. However, Suzuki et al. have reported that electron irradiation of 20 eV, which is much lower than 86 keV, damages SWCNTs and alters their electrical properties [20]. The vacancy defects introduced by these various processes are energetically unstable, and structures such as mono-vacancies and di-vacancies, which lack one and two carbon atoms, respectively, often gain a small amount of energy and change to a metastable structure [21]. The metastable structure and five-, seven-, and eight-membered rings are introduced by thermal rearrangement. In this paper, five- and seven-membered rings are referred to as “non-six-membered ring defects.” 

### 1.3. Non-Six-Membered Ring Defects and Conductivity

**Figure 1:**
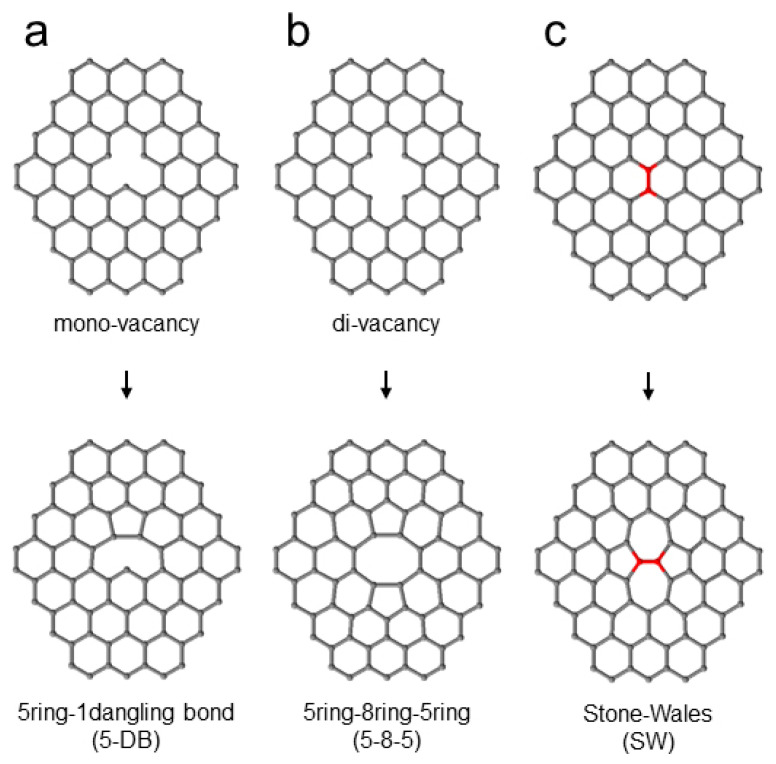
Schematic diagrams of non-six-membered defects. (**a**) Mono-vacancy defect changing to a combination of one five-membered ring and one dangling bond (5-1DB). (**b**) Di-vacancy defect changing to a combination of two five-membered rings and one eight-membered ring (5-8-5). (**c**) Stone–Wales (SW) defects (red parts).

Five- and seven-membered rings in non-six-membered ring defects are the most easily introduced defects [15]. For example, the formation energy of single 5–7 defects or that of Stone–Wales (SW) 5-7-7-5 defects is 3.4 eV, whereas the formation energies of the mono-vacancy defects and di-vacancy defects are 7.4 and 5.0 eV, respectively. The rearrangement of vacancy defects by heat is one of the most common methods for introducing non-six-membered ring defects [22]. For instance, a mono-vacancy defect changes to a combination of one five-membered ring and one dangling bond (5-1DB in Figure 1a), and a di-vacancy defect changes to a combination of two five-membered rings and one eight-membered ring (5-8-5 in Figure 1b), introducing a non-six-membered ring defect by forming a metastable structure [23,24]. Compared with that obtained by the rearrangement of vacancy defects with a few atoms missing, the carbon framework obtained by the rearrangement of vacancy defects with dozens of missing atoms is highly complex. Charlier et al. reported a relationship between the number of carbon atoms lost by electron irradiation and the structure obtained by the rearrangement [23] and predicted a decrease in SWCNT diameter and the collapse of the carbon framework as the number of carbon atoms lost increased. In addition, a 90-degree rotation of the carbon bond (C=C) changes a four-six-membered ring into two five-membered and two seven-membered rings, which are collectively known as an SW defect (Figure 1c) [25]. SW defects are introduced when carbon bonds become unstable because of electron irradiation [26]. 

The presence of vacancy defects reduces the electrical conductivity of SWCNTs because they scatter electrons and block conductive paths. Intriguingly, the DOS of SWCNTs with introduced non-six-membered ring defects has been reported theoretically [27]. Defect-free and highly crystalline SWCNTs possess a DOS that exhibits Van Hove singularity derived from their one-dimensional structure. However, the introduction of non-six-membered ring defects into SWCNTs causes two changes: the disappearance of the band gap and the presence of DOS at the Fermi level (Figure 5d in [27]). Thus, the introduction of non-six-membered ring defects may improve the conductivity of the SWCNTs.

### 1.4. Introduction of Non-Six-Membered Rings

To investigate the effect of non-six-membered ring defects on the electrical properties of SWCNTs, samples with a high concentration of non-six-membered ring defects are necessary. Two processes are effective for introducing non-six-membered ring defects: “introduction of vacancy defects” and “rearrangement of vacancy defects by thermal treatment.” When using ball milling or wet oxidation, such as nitric acid, to introduce vacancy defects, the surfaces of the SWCNTs partially collapse, leading to the introduction of large vacancy defects and cutting of the nanotubes [18,28], and the nanotube structure cannot be maintained during thermal rearrangement. Therefore, to introduce non-six-membered ring defects while retaining the structure of SWCNTs, gas-phase treatment, which is expected to introduce small-size vacancy defects, is preferable.

Previously, we have introduced vacancy defects into SWCNTs via a fluorination–defluorination process [29,30]. In this method, the carbon framework is rearranged and vacancy defects are formed while fluorinated SWCNTs are heated at temperatures of 300–600 °C, and the fluorine groups are detached in the form of volatile carbon fluorides along with carbon atoms from the nanotube framework. Fluorination has several advantages, such as uniform introduction of fluorine groups on the SWCNTs surface and easy control of the number of introduced fluorine groups by varying reaction temperature and time [31,32]. Given that the number of non-six-membered ring defects can be controlled by adjusting the number of fluorine groups, the fluorination–defluorination process is expected to be a low-energy and highly efficient method for introducing non-six-membered ring defects. 

Here, we prepared defect-introduced SWCNTs via the fluorination–defluorination process from SWCNTs fluorinated at a temperature of 25 °C for different reaction times and evaluated their structure. We measured the conductivity of the defect-introduced SWCNTs by operating a temperature program and discussed the presence of non-six-membered ring defects into the nanotube. We successfully synthesized F-SWCNTs with a low fluorination degree. Structural analysis of the defect-introduced SWCNT samples using X-ray photoelectron spectroscopy (XPS), Raman, high-resolution TEM (HRTEM), and visible/near-infrared (vis-NIR) spectroscopy did not reveal the presence of non-six-membered ring defects in the SWCNTs but revealed the introduction of vacancy defects. Meanwhile, conductivity measurements performed by operating a temperature program showed that defect-introduced SWCNTs from F-SWCNTs reacted for 3 min exhibited a conductivity decrease owing to the adsorption of water molecules to non-six-membered ring defects, and we observed the possibility that non-six-membered ring defects were introduced into the nanotubes using the fluorination–defluorination process.

## 2. Materials and Methods

### 2.1. Preparation of Purified SWCNTs

SWCNTs were prepared using an arc-discharge method. The synthesis procedure has been detailed in previous reports [31,32,33]. Soot was synthesized by arc discharge between a pure graphite cathode and anode rods and catalyzed by Fe, Ni, and S powders. To remove amorphous carbon from the soot, the soot was successively oxidized at 450 °C for 30 min and 500 °C for 30 min under an air atmosphere using a muffle furnace (FO300, Yamato Scientific Co., Ltd., Tokyo, Japan). The air-oxidized soot was immersed in a hydrochloric acid solution (6.0 mol L^−1^) to remove any metal particles, and the resulting suspension was filtered using a polytetrafluoroethylene (PTFE) membrane filter with a pore diameter of 3.0 μm (ADVANTEC, Tokyo, Japan). The filter cake was washed with deionized water until the filtrate was pH-neutral, dispersed in deionized water, and freeze-dried. Finally, the freeze-dried sample was annealed under vacuum (1.0 × 10^−5^ Pa) at 1200 °C for 3 h to remove oxygen-containing functional groups, such as carboxyl and ketone groups [34,35,36]. Consequently, purified SWCNTs (P-SWCNTs) were obtained. The impurities in the P-SWCNTs were graphite (1.5 wt.%), Si (0.7 wt.%), Fe (0.6 wt.%), and Ni (0.7 wt.%). Fe and Ni existed as Fe*_x_*Ni*_y_*C*_z_* nanoparticles in thick graphite capsules.

### 2.2. Preparation of Samples via the Fluorination–Defluorination Process

The P-SWCNTs in a PTFE cell set at the center of an electronic tubule furnace (ARF-30K, Asahi Rika Seisakusho Co., Ltd., Chiba, Japan) were fluorinated at 25 °C for given reaction times (3 min and 4 h) using a mixture of F_2_ (20%) and N_2_ (80%) at a flow rate of 25 mL min^−1^. SWCNTs fluorinated for 3 min and 4 h are referred to as “F-RT-3m” and “F-RT-4h,” respectively. Each F-SWCNT was annealed under vacuum (1.0 × 10^−5^ Pa) at 1200 °C for 3 h, and the obtained samples were denoted by “deF-RT-3m” and “deF-RT-4h,” respectively.

### 2.3. Characterization

XPS was performed using a K-Alpha^+^ system (Thermo Fisher Scientific Inc., Waltham, MA, USA) with a monochromatic Al Kα X-ray source. The samples for XPS measurements were heated at 110 °C for 1 h under vacuum (1.0 × 10^−3^ Pa) to remove the adsorbed water molecules. The morphologies of the samples were observed using scanning electron microscopy (SEM; SU-8000, Hitachi High-Tech Corporation, Tokyo, Japan) and HRTEM (HF-2000, Hitachi High-Tech Corporation, Japan). Raman scattering spectroscopy (Jobin Yvon T64000, Horiba Co., Ltd., Kyoto, Japan) was performed in the backscattering mode at room temperature using a diode-pumped solid-state laser (Cobolt Blues^™^, Cobolt, Stockholm, Sweden) with an excitation wavelength of 473 nm to evaluate the vibrational modes of the nanotubes. The vis-NIR transmission absorbance spectra of the thin films were measured using a UV-visible spectrophotometer (U-3900, Hitachi High-Tech Corporation, Tokyo, Japan) and an NIR spectrophotometer (Frontier MIR/NIR, PerkinElmer Co., Ltd., Waltham, MA, USA). The permeable thin films were prepared as follows: a suspension was prepared by dispersing the sample (5 mg) in ethanol (50 mL) and sonicating for 15 min. The suspension was uniformly sprayed onto a glass slide using a spray-coating method.

### 2.4. Conductivity Measurement by Operating a Temperature Program

The conductivities of the samples were measured using the Van der Pauw method via a resistivity/Hall measuring system (RESITEST 8200, TOYO Corporation, Tokyo, Japan). SWCNT films (diameter: 15 mm, thickness: 100 μm) were prepared by vacuum filtration of ethanol-dispersed SWCNTs. The prepared films were dried up in an oven at 60 °C in air for at least 12 h. Notably, that this drying condition does not allow for complete dehydration. Each film was placed on a ceramic heater, and a quartz glass plate with embedded gold electrodes was placed on the film and fixed with pins. After the sample was placed in the vacuum chamber, the exhaust pressure was evacuated to 1.0 × 10^−3^ Pa. The conductivity of sample films was measured by operating a temperature program of 25 °C → 500 °C → 25 °C (rate of temperature rise and fall: 10 °C min^−1^). The conductivity measurements were repeated five-times for each sample.

## 3. Results and Discussion

### 3.1. Structural Characterization

**Figure 2:**
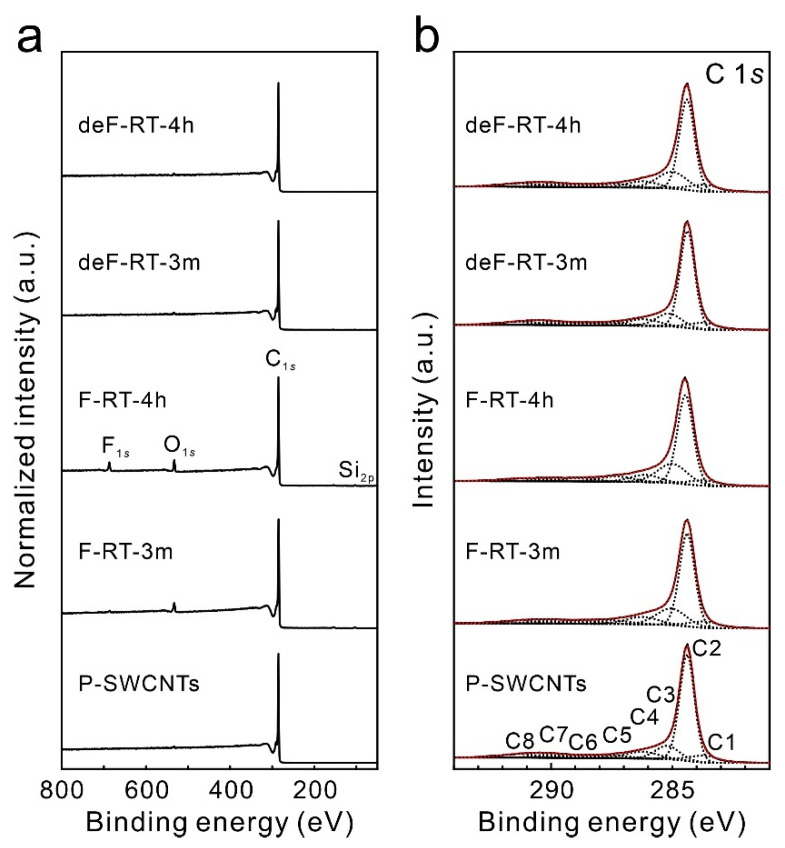
(**a**) X-ray photoelectron spectroscopy (XPS) wide and (**b**) XPS C 1*s* narrow spectra of purified, fluorinated, and defluorinated single-walled carbon nanotubes (P-SWCNTs, F-SWCNTs, and deF-SWCNTs, respectively). The black and red dotted lines represent deconvoluted peaks and fitted curves, respectively.

The XPS wide spectra, XPS C 1*s* and F 1*s* narrow spectra, and elemental concentrations of P-SWCNTs, F-SWCNTs, and deF-SWCNTs are shown in Figure 2 and Table 1. The intensity of each XPS spectrum was normalized to the peak intensity of C 1*s* for each sample. Silicon and oxygen were detected in all samples (Figure 2a). Figure S1a shows the XPS Si 2*p* spectrum of each sample. The peak at 101 eV is attributed to the binding energy of the Si-C bond [37,38], the peak at 102 eV to the Si-O/Si-O-C bond [39,40] and the -Si-O-F bond [41], and the peaks from 103 eV to 105 eV to the binding energy of SiO_2_ [42]. The concentrations of Si in deF-RT-3m and deF-RT-4h were lower than those in F-RT-3m and F-RT-4h. This phenomenon can be attributed to the decomposition of Si-O-F groups in the silicon compound by thermal treatment. We speculate that the Si impurities are related to the equipment used to synthesize SWCNTs. A quartz glass tube was used as an insulator of the arc-discharged anode and was considered to melt and vaporize owing to the high temperatures caused by arc discharge, resulting in the mixing of silicon compounds into the soot.

For oxygen (Figure S1b), the peaks at approximately 530–532 eV were assigned to the binding energy of the C=O bond derived from carbonyl and quinone groups [43], while those at approximately 533–534 eV were ascribed to the binding energy of the C-O bond [42] and SiO_2_ [42]. Meanwhile, the peaks between 533 and 536 eV are the binding energies of adsorbed water, and the binding energy of multilayer water adsorption appears on the high-energy side [44,45]. The oxyfluorinated SWCNTs with oxygen-containing fluorine groups can be synthesized by refluxing fluorinated SWCNTs in aqueous solution [46]. Accordingly, considering the high concentration of oxygen atoms in the fluorinated SWCNTs in this study, it is more likely that the fluorinated samples were exposed to water and oxygen molecules and were partially oxidized to contain O-C-F functional groups.

Figure 2b shows the XPS C 1*s* narrow spectrum of each sample, which is deconvoluted into eight peaks. The C1 peak (283.0 eV) is assigned to carbon atoms bonded to silicon atoms [37], while the C2 peak (284.3 eV) is associated with carbon atoms in *sp*^2^-hybridized graphitic structures [47]. The C3 peak (285.0 eV) can be assigned to *sp*^2^-hybridized carbon atoms bonded to *sp*^3^-hybridized carbon atoms that are bonded to functional groups. When the *sp*^3^-hybridized carbon atom (*sp*^3^-C) in carbon materials is charged by the break of the electron pathway between *sp*^2^-hybridized carbon atoms (*sp*^2^-C) and *sp*^3^-C, the peak of *sp*^3^-C undergoes a positive shift relative to the position of the *sp*^2^-C peak [48]. In our samples, the F-SWCNTs contained *sp*^3^-C bonded with fluorine groups (*sp*^3^-C-F). When the electron pathway between *sp*^2^-C and *sp*^3^-C-F is disconnected, *sp*^3^-C is charged and experiences a positive shift relative to the position of the *sp*^2^-C peak. Moreover, the C4 peak (286.1 eV) is associated with carbon atoms in the hydroxyl groups [49]. The C5 peak (287.2 eV) is assigned to carbon atoms bound to oxygen atoms in ketone and quinone groups [50] and/or *sp*^2^-C bound to intercalated fluorine atoms, which is termed the “semi-ionic bond” [50]. The C6 peak (288.8 eV) is ascribed to carbon atoms bound to oxygen atoms in ester and carboxyl groups [49] and/or *sp*^2^-C bound to fluorine groups [50]. The C7 peak (289.3–290.2 eV) is assigned to -CF- and -CF_2_- bonds [50], and the C8 peak (291.3 eV) is derived from -CF_3_ bonds [50]. In addition, C7 and C8 correspond to the characteristic vibration modes of the carbon atoms in aromatic structures. The functionalized groups are difficult to identify based only on the XPS C 1*s* spectrum because the chemical shifts of the oxygen-containing functional groups and fluorine groups are at a similar peak position.

**Figure 3:**
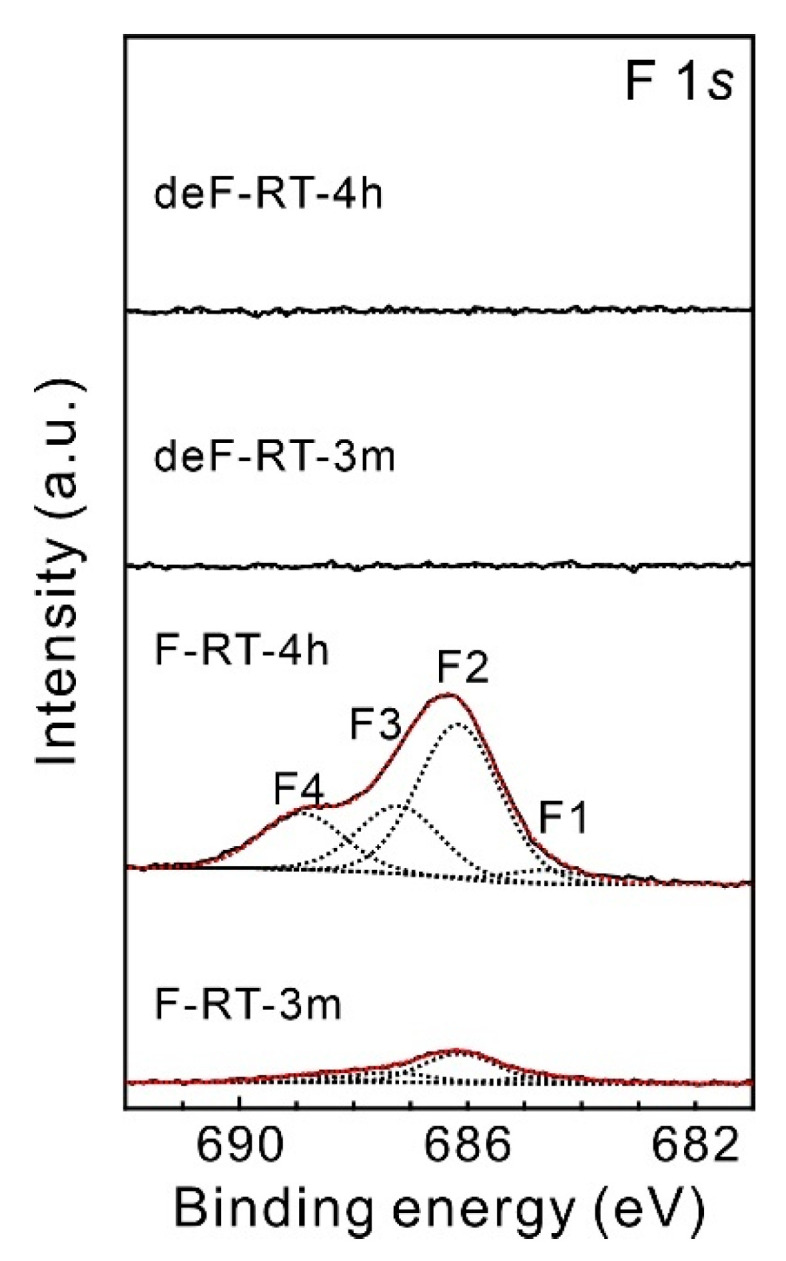
XPS F 1*s* narrow spectra of F-SWCNTs and deF-SWCNTs. The black and red dotted lines represent deconvoluted peaks and fitted curves, respectively.

The XPS F 1*s* narrow spectrum of each sample was deconvoluted into four peaks (Figure 3). The F1 peak (684.7 eV) in the spectra of the F-SWCNTs corresponds to the fluorine groups characterized by ionic fluorine–carbon bonding, which are associated with graphite fluorides with low degrees of fluorination (CF*_x_* with *x* < 0.05) [51,52] as well as adsorbed or entrapped fluorine [53]. The F2 peak (686.1 eV) corresponds to fluorine groups characterized by ionic fluorine–carbon bonding, which are associated with graphite fluorides with high degrees of fluorination (CF*_x_*, with 0.05 < *x* < 0.28) [52,54]. The F3 (~687.3 eV) and F4 (~688.8 eV) peaks are associated with fluorine groups characterized by semi-covalent and covalent bonding, respectively [51]. The peak at 688.8 eV is also assigned to O-C-F groups [46,55]. Meanwhile, the existence of silicon oxyfluoride species such as Si_3-x_SiF_x_ surface groups with binding energy of 686.0 eV and (SiO)_3-x_SiF_x_ with binding energy of 689.5 eV is also suggested [56,57]. According to An et al., F-SWCNTs exhibit ionic-bonding characteristics at low concentrations and covalent-bonding characteristics at high concentrations [58]. In relation to this phenomenon, Tressaud et al. reported that adjacent fluorine groups on a nanotube frame, fluorine groups in the collective state, shift the XPS F 1*s* peaks to higher binding energies because each highly polar C-F bond withdraws electrons from the neighboring C-F bonds [50]. In our previous report on the modification of SWCNTs by radical fluorine atoms using gaseous XeF_2_, as the number of collective fluorine groups increases, the proportion of C-F bonds with a high binding energy increases [30]. In F-RT-4h, the peak at 689.0 eV was larger than that at 687.2 eV, indicating a larger percentage of collective fluorine groups. Meanwhile, after thermal treatment at 1200 °C for 3 h under vacuum, minimal fluorine was detected, indicating that the samples were defluorinated. 

Based on the concentrations of fluorine and carbon atoms obtained from the XPS analysis, the F/C ratios of F-RT-3m and F-RT-4h were 0.006 and 0.031, respectively, indicating that more fluorine groups were modified in F-RT-4h than in F-RT-3m (Table 1). However, their accurate F/C ratios cannot be estimated owing to the presence of overlapping bonds such as Si-O-F, C-O-F, and C-F bonds. Therefore, the actual F/C ratios of the fluorinated samples are smaller than the estimated F/C ratios.

**Figure 4:**
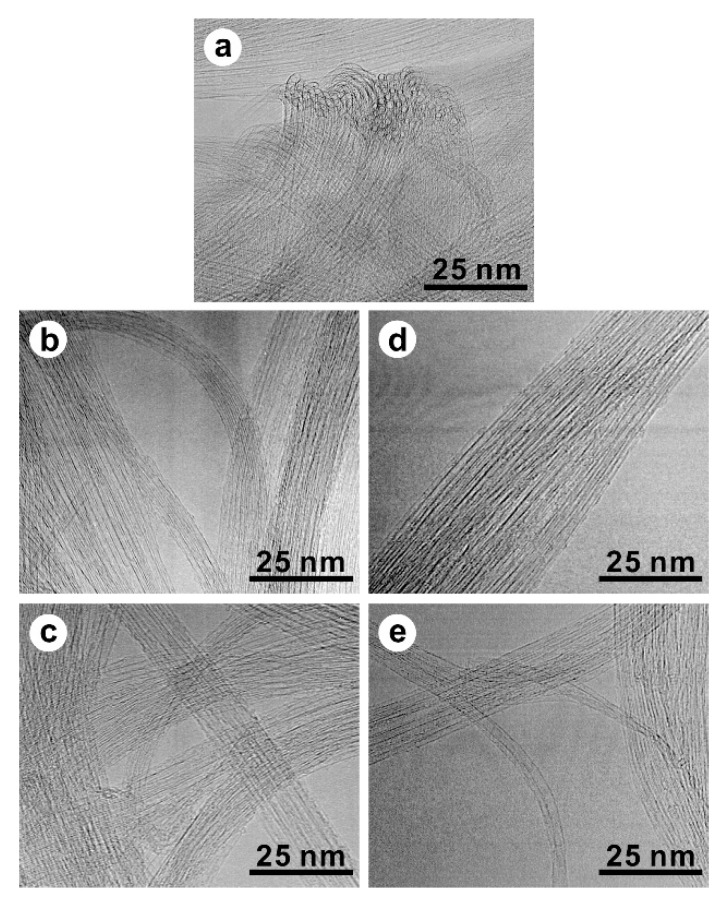
High-resolution transmission electron microscopy (HRTEM) images of the (**a**) P-SWCNTs, (**b**) F-RT-3m, (**c**) F-RT-4h, (**d**) deF-RT-3m, and (**e**) deF-RT-4h samples.

In the HRTEM images of F-RT-3m and F-RT-4h, the nanotube structure was observed as clearly as that in the P-SWCNTs (Figure 4a–c). The diameter of the SWCNTs was 1.3 to 1.5 nm. SWCNTs formed a bundle structure with a diameter of approximately 30 nm, and no change in the bundle structure was observed after fluorination. The deF-RT-3m and deF-RT-4h retained the nanotube framework while maintaining the bundle structure, and their TEM images were not significantly different from those of the P-SWCNTs, F-RT-3m, and F-RT-4h (Figure 4d,e).

**Figure 5:**
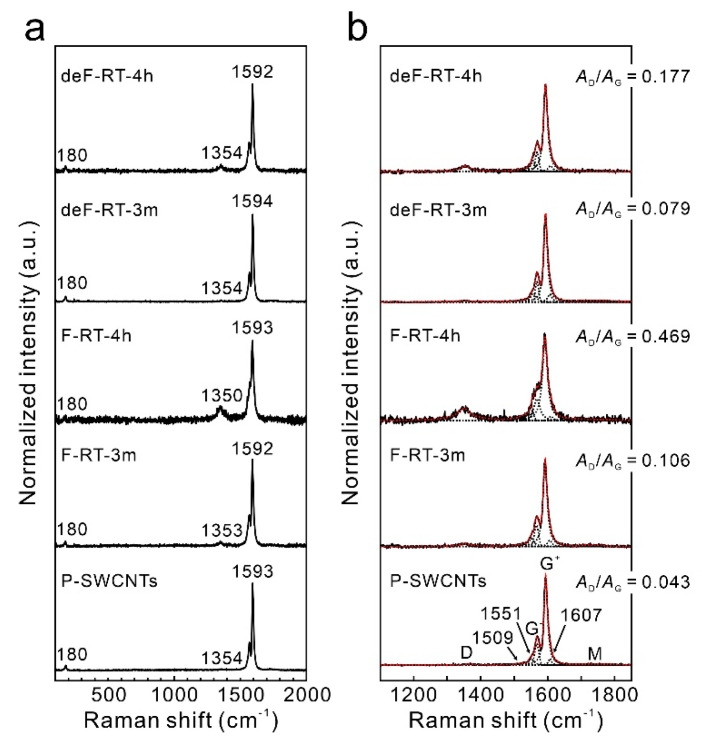
(**a**) Raman spectra of all samples in the range of 100–1850 cm^−1^. (**b**) Deconvoluted Raman spectra of all samples in the range 1100–1850 cm^−1^. The black and red dotted lines represent deconvoluted peaks and fitted curves, respectively.

A peak appeared at 180 cm^−1^ in the radial breathing mode (RBM) region of the Raman scattering spectrum of the P-SWCNTs (Figure 5a). The diameter of the SWCNTs can be approximated using the following equation considering the bundle structure of the nanotubes [59]:

*ω* (cm^−1^) = 217.8/*d* (nm) + 15.7

Therefore, the positions of the highest-intensity RBM bands correspond to nanotube diameters of approximately 1.33 nm. SWCNTs with RBM intensity enhanced by the resonance Raman effect when a 473 nm (2.62 eV) laser is used are semiconducting SWCNTs. As the degree of fluorination increased, the RBM intensity decreased, and the D-band intensity increased in the F-SWCNT samples (Figure 5a). By contrast, for the deF-SWCNT samples, the RBM and D-band intensities returned to near their original intensities.

The Raman spectra (1100–1850 cm^−1^) of the samples were deconvoluted into the following peaks (Figure 5b): D band (1350 cm^−1^), E2 symmetry phonons (1510 and 1607 cm^−1^), Breit–Wigner–Fano lineshape (1550 cm^−1^), G (G^−^ (1567 cm^−1^) and G^+^ bands (1592 cm^−1^)), and M (M^−^ (1730 cm^−1^) and M^+^ bands (1780 cm^−1^)), which are attributed to the overtones of out-of-plane and infrared-active modes at 867 cm^−1^ in graphite [60]. The *A*_D_/*A*_G_ ratio in Figure 5b is the ratio of the integrated intensity of the D band to that of the G band and represents the relative number of defects. The *A*_D_/*A*_G_ of F-RT-4h (0.419 ± 0.038) was larger than that of F-RT-3m (0.106 ± 0.026), indicating that the fluorine groups in F-RT-4h were greater than those in F-RT-3m. By contrast, the *A*_D_/*A*_G_ of deF-RT-4h (0.177 ± 0.018) was larger than that of deF-RT-3m (0.079 ± 0.005), indicating that deF-RT-4h had a larger number of defects than deF-RT-3m. 

**Figure 6:**
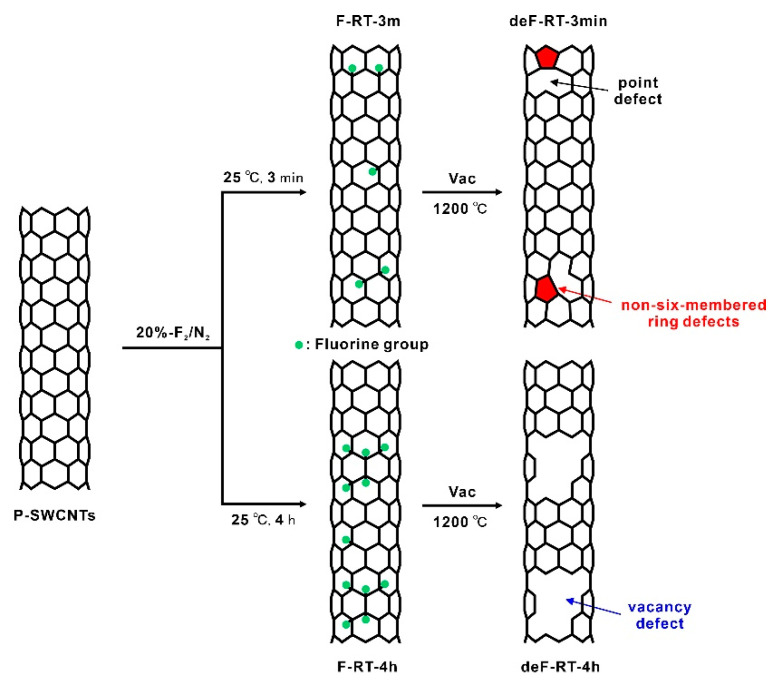
Schematic illustration of the defluorinated SWCNTs prepared by the fluorination–defluorination process.

In detail, we considered the number of defects in P-SWCNTs, F-RT-3m, F-RT-4h, deF-RT-3m, and deF-RT-4h. The average lengths of P-SWCNTs, F-RT-3m, F-RT-4h, deF-RT-3m, and deF-RT-4h were 3.06 (±0.50), 2.75 (±0.60), 2.78 (±0.76), 3.09 (±0.66), and 2.96 (±0.59) μm, respectively, with no significant variation (Table 2, length distribution in Figure S2). When F-SWCNTs with a fluorination degree of CF_0.4~0.5_ are defluorinated, the fluorine groups are detached along with carbon atoms from the nanotube framework in the form of carbon fluorides, resulting in the cutting of nanotubes [29,61]. In this study, the ultra-low F-SWCNTs were not cut by defluorination, suggesting that point and vacancy defects were formed in the nanotubes (Figure 6). The crystallite size (*L*_a_) of each sample was estimated using the following equation [62]:

$$L_{a}\;\left(µm\right)=\frac{560\times 10^{-3}}{E_{l}{}^{4}\times \left(A_{D}/A_{G}\right)}$$

where *E_l_* is the excitation laser energy (eV), *A*_D_ is the integrated intensity of the D-band, and *A*_G_ is the integrated intensity of the G-band. The *L*_a_ of each sample increased in the order of F-RT-4h < deF-RT-4h < F-RT-3m < deF-RT-3m < P-SWCNTs, indicating that the larger the number of defects, the smaller the *L*_a_ (Table 2). Isolated fluorine groups functionalized to SWCNTs are desorbed without the carbon atoms of nanotubes when heated in vacuum or in an inert gas flow [30]. Therefore, the *L*_a_ size of the defluorinated samples is larger than that of the fluorinated samples after heat treatment because the carbon atoms with *sp*^3^ hybridized orbitals, to which the isolated fluorine groups were bonded, change to carbon atoms with *sp*^2^ hybridized orbitals (Figure 6). The apparent number of defects *L*/*L*_a_ was estimated as the number of defects [30]. The word “apparent” is used because *L*/*L*_a_, which estimates the number of point defects on a line by calculation, does not reflect vacancy defects. Based on the *L*/*L*_a_ of each sample, F-RT-4h had the most defects among all samples (Table 2). Furthermore, 1/*L*_a_, the apparent number of defects per unit of nanotube length (µm), increased in the order of P-SWCNTs < deF-RT-3m < F-RT-3m < deF-RT-4h < F-RT-4h. The 1/*L*_a_ ratio did not show the type of defect. The presence of the edge of the vacancy defect cannot be recognized by Raman scattering spectroscopy because the vibration (1620 cm^−1^) originating from the edge of graphene does not appear [63]. However, the XPS results show that the number of collective fluorine groups is higher in deF-RT-4h and that more vacancy defects may be present in deF-RT-4h than in deF-RT-3m (Figure 6).

### 3.2. Conductivity

**Figure 7:**
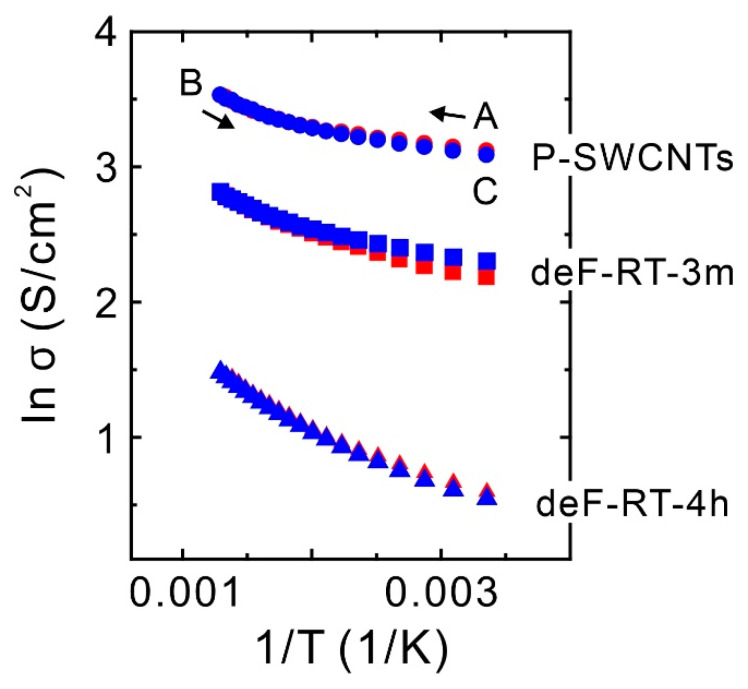
Electrical conductivity of the P-SWCNT, deF-RT-3m, and deF-RT-4h films as a function of temperature. Red and blue represent the conductivities from A to B and from B to C, respectively.

Figure 7 shows a plot of conductivity versus temperature for the P-SWCNT, deF-RT-3m, and deF-RT-4h films. Point A denotes the conductivity at 25 °C before heating was applied, and the conductivity was measured while temperature increased from 25 °C (point A) to 500 °C (point B) and decreased from 500 °C (point B) to 25 °C (point C). The conductivities of all samples increased with increasing temperature. One assumption is that the influence of semiconducting SWCNTs, which account for 90% of the SWCNTs used in this study, is dominant in the electrical conductivity. In addition, a slight difference appeared in the conductivity between the temperature increase from 25 °C to 200 °C and the temperature decline from 200 °C to 25 °C in all the samples. In P-SWCNTs and deF-RT-4h, the conductivity during the temperature increase was slightly larger than that during the temperature decrease. Meanwhile, the opposite was observed for deF-RT-3m; that is, the conductivity of the deF-RT-3m during temperature decline was slightly larger than that during the temperature rise. Table 3 shows the conductivity at 25 °C of each sample before and after applying temperature. For P-SWCNTs and deF-RT-4h, the conductivity before heat application was slightly higher than that after application. However, for deF-RT-3m, the conductivity before heating was slightly smaller than that after heating. The behavior of the change in conductivity at 25 °C before and after heat application was found to be reproducible. Furthermore, for all samples, the temperature-dependent measurements after the second cycle showed almost the same conductivity values as that between points B and C in the first cycle. 

**Figure 8:**
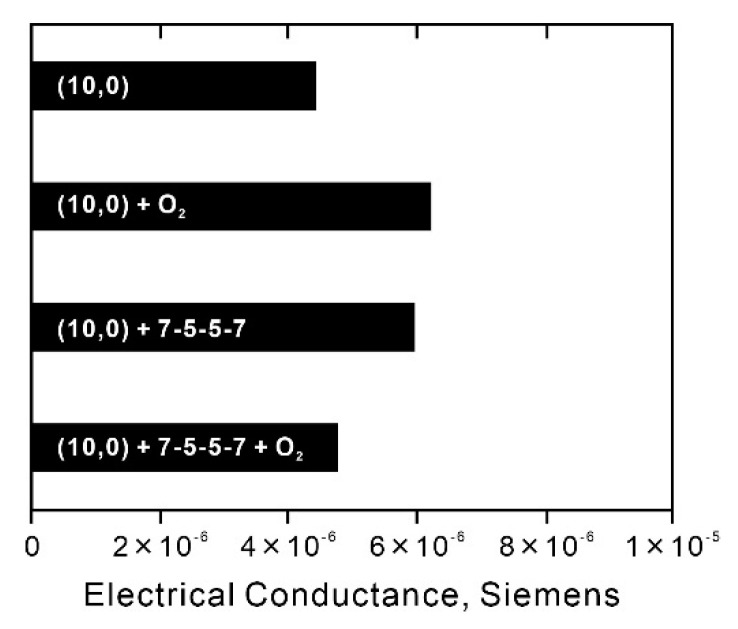
Effect of O_2_ adsorbates and 7-5-5-7 topological defects on the electrical conductance of semiconducting zig-zag (10, 0) SWCNTs. Reprinted with permission from ref. [67]. Copyright 2023 Elsevier, Ltd.

The above phenomenon is considered to be related to oxygen and/or water molecules adsorbed on the nanotube surface. Zahab et al. reported that a few water molecules adsorb onto the basal plane of SWCNTs and act as acceptors, increasing the conductivity of water-absorbed SWCNTs that behave as *p*-type semiconductors [64]. Moreover, when oxygen molecules are physisorbed onto SWCNTs, the conductivity of the SWCNTs increases as a *p*-type semiconductor, and when oxygen molecules are desorbed from the SWCNTs, their conductivity decreases [65,66]. Furthermore, Grujicic et al. reported in their theoretical calculations that when 5-7-7-5 (pentagon–heptagon–heptagon–pentagon)-type non-six-membered ring defects are present in semiconducting zigzag (10,0)-SWCNTs, the chemisorption of oxygen molecules on the non-six-membered ring defects results in a decrease in conductivity [67]. This outcome indicates that the effect of non-six-membered ring defects, which theoretically contribute to the increased conductivity of semiconducting SWCNTs, is counteracted by chemically adsorbed oxygen molecules (Figure 8). Considering the phenomenon of conductivity change in this study with reference to these reports, we think that water molecules acting as acceptors in the P-SWCNTs increase the conductivity by adsorbing onto the basal plane and that the conductivity decreases after desorption. In deF-RT-3m, because the number of non-six-membered ring defects to nanotube basal planes increases, the conductivity is assumed to be reduced by the water molecules adsorbed on the non-six-membered ring defects and increased by desorption. Meanwhile, in deF-RT-4h, the number of non-six-membered ring defects decreases and the nanotubes are mainly composed of basal planes with vacancy defects. Water molecules adsorb on the basal surfaces, and the conductivity of deF-RT-4h increases and then decreases after desorption, similar to the conductivity behavior of P-SWCNTs. Thus, conductivity measurements indicate that non-six-membered ring defects may be present in deF-RT-3m. 

**Figure 9:**
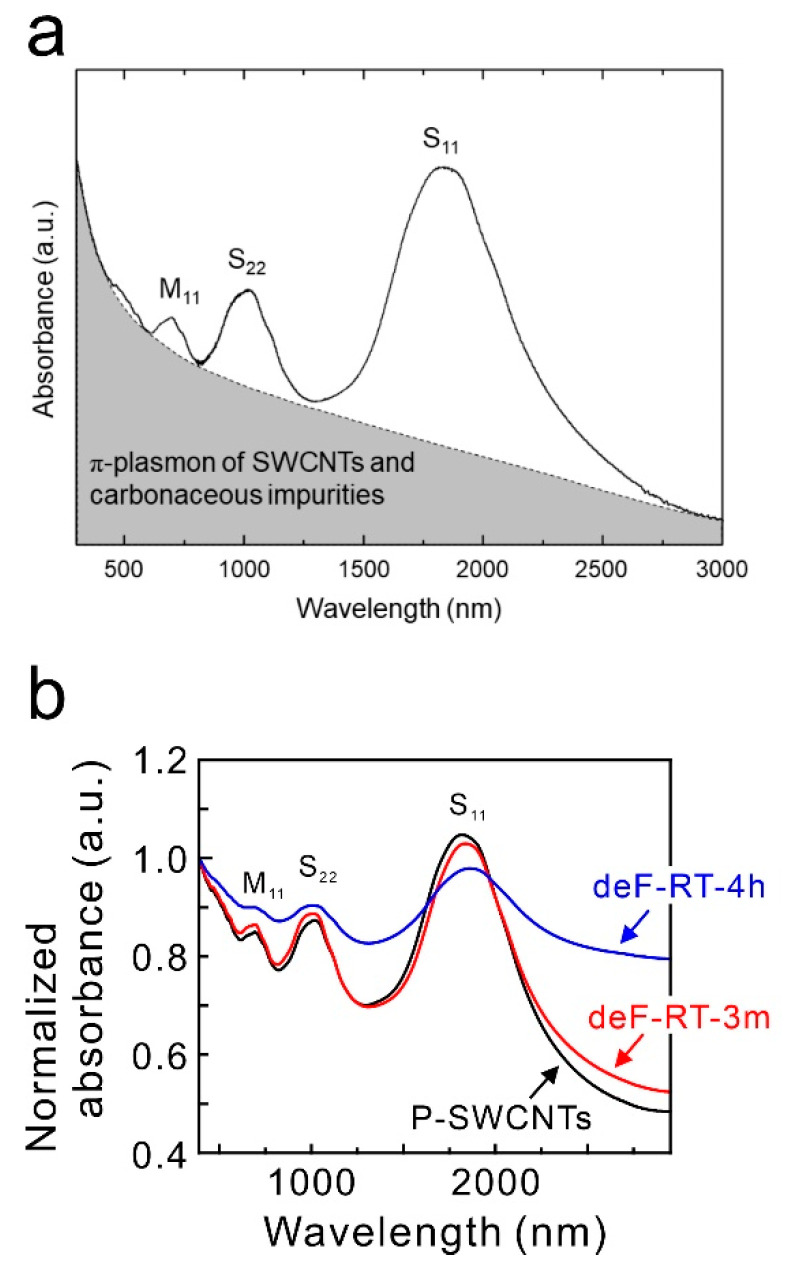
(**a**) Visible/near-infrared (Vis-NIR) spectrum of typical SWCNTs synthesized by the arc discharge method. (**b**) Vis-NIR spectra of P-SWCNTs, deF-RT-3m, and deF-RT-4h.

Theoretical calculations reported by Grujicic et al. have shown that the conductivity of semiconducting (10,0)-SWCNTs with non-six-membered ring defects without chemisorption of oxygen molecules is higher than that of untreated semiconducting (10,0)-SWCNTs (Figure 8) [67]. In contrast to their theoretical model, this study shows that the conductivity of deF-RT-3m without oxygen molecules, which may have non-six-membered ring defects, is lower than that of P-SWCNTs. Carlsson et al. computationally investigated the relationship between the formation energy of defects and the chirality of nanotubes and reported that defects are more likely to form in metallic SWCNTs than in semiconducting SWCNTs [68]. Tang et al. evaluated the ratio of metallic to semiconducting nanotubes in defluorinated SWCNTs (deF-SWCNTs) using Raman scattering spectroscopy and vis-NIR absorbance measurements and reported that defluorination reduces the ratio of metallic SWCNTs with small diameters (1.1 nm) [69]. Whether this decrease in conductivity of deF-RT-3m is due to vacancy defects or the destruction of metallic nanotubes should be determined. To evaluate the ratio of metallic to semiconducting nanotubes in deF-RT-3m, the vis-NIR spectra of the samples were measured. Figure 9a shows the vis-NIR spectrum of a typical SWCNT thin film synthesized using the arc discharge method. S_11_ and S_22_ are the first and second interband transitions of semiconducting SWCNTs, respectively, and M_11_ is the first interband transition of metallic SWCNTs. The grey part of the spectrum represents π-plasmon absorption from both SWCNTs and carbonaceous impurities [70]. The ratio of metallic nanotubes to semiconducting nanotubes in SWCNTs can be evaluated by calculating the integral intensity ratio of the absorption peak (M_11_) derived from metallic SWCNTs to the absorption peak (S_11_ or S_22_) from semiconducting SWCNTs in the vis-NIR spectrum of SWCNTs [70]. Here, the ratio of metallic nanotubes to semiconducting nanotubes was evaluated using the M_11_ and S_22_ adsorption peaks. The background was subtracted from each absorbance peak, and the ratio of the integrated intensities was calculated. Figure 9b shows the vis-NIR spectra of P-SWCNTs, deF-RT-3m, and deF-RT-4h. The results show that the percentages of metallic nanotubes in the P-SWCNTs and deF-RT-3m are approximately 12–13% and that the metallic nanotube ratios of the two samples have no significant difference (Table 4). Meanwhile, the percentage of metallic nanotubes in deF-RT-4h is approximately 7%, suggesting that vacancy defects preferentially occur in metallic nanotubes rather than in semiconducting ones. In addition, the background of the vis-NIR spectrum increases in the order of P-SWCNT ≈ deF-RT-3m < deF-RT-4h. The increase in the background of the vis-NIR spectrum of deF-RT-4h is attributed to an increase in the absorption of π-plasmons due to vacancy defects in the nanotubes. 

From the results of Raman scattering spectroscopy and Vis-NIR absorption spectroscopy, we conclude that the decrease in the conductivity of deF-RT-3m is due to the presence of vacancy defects rather than a decrease in the ratio of metallic nanotubes. To achieve conductivity beyond that of P-SWCNTs, preparing SWCNTs with higher relative concentrations of non-six-membered ring defects is necessary. Thus, optimization of fluorination conditions, such as use of gaseous XeF_2_ to modify F-SWCNTs with isolated fluorine groups [30,71], is expected to be effective. 

## 4. Conclusions

The introduction of non-six-membered ring defects, such as five- and seven-membered rings, into SWCNTs is expected to enhance conductivity by increasing the DOS at the Fermi energy level. However, no preparation method has been established to efficiently introduce non-six-membered ring defects into SWCNTs. In this study, we attempted to introduce non-six-membered ring defects into SWCNTs by defect rearrangement of the nanotube framework using a fluorination–defluorination process to prepare SWCNTs with non-six-membered ring defects. Here, we prepared defect-introduced SWCNTs from SWCNTs fluorinated at 25 °C for different reaction times via the fluorination–defluorination process, evaluated their structure, and measured the conductivity of the defect-introduced SWCNTs.

We succeeded in synthesizing F-SWCNTs with a low degree of fluorination. Structural analysis of the deF-SWCNT samples using XPS, Raman, HRTEM, and vis-NIR did not reveal the presence of non-six-membered ring defects in the SWCNTs but showed the introduction of vacancy defects in the nanotubes. Meanwhile, conductivity measurements performed by operating a temperature program showed that deF-RT-3m exhibited decreased conductivity due to the adsorption of water molecules to non-six-membered ring defects, and we found the possibility that non-six-membered ring defects were introduced into deF-RT-3m. However, a decrease in conductivity due to the introduction of vacancy defects associated with the collective desorption of fluorine groups has also been suggested. To prepare SWCNTs with higher relative concentrations of non-six-membered ring defects, optimization of fluorination conditions, such as using gaseous XeF_2_ to prepare F-SWCNTs with isolated fluorine groups, is expected to be effective. 

In the future, if we can identify the types of non-six-membered ring defects introduced by a fluorination–defluorination process and measure the DOS of these defects via surface analysis using a scanning tunneling microscope, more detailed information on the electrical properties of non-six-membered ring defects can be obtained.

## Figures and Tables

**Table 1:** Elemental concentrations of P-SWCNTs, F-SWCNTs, and deF-SWCNTs. The binding energies and chemical compositions of each sample were determined at three different positions to obtain the average value.

Samples	Chemical Composition (at.%)				Component of Fluorine Species (at.%)			
	C	O	F	Si	F1	F2	F3	F4
P-SWCNTs	99.34	0.42	-	0.24	-	-	-	-
F-RT-3m	94.68	3.77	0.53	1.02	0.07	0.28	0.11	0.07
F-RT-4h	91.78	4.37	2.88	0.95	0.13	1.51	0.67	0.57
deF-RT-3m	99.00	0.65	0.00	0.35	-	-	-	-
deF-RT-4h	99.09	0.61	0.00	0.30	-	-	-	-

**Table 2:** Average nanotube length, crystallite size, apparent number of defects, and apparent linear density of defects per unit nanotube length of P-SWCNTs, deF-RT-3m, and deF-RT-4h. The Raman spectra of each sample were recorded at five different positions.

Samples	Length (μm)	Crystallite Size *L*_a_ (μm)	Apparent Number of Defects *L*/*L*_a_	Apparent Linear Density of Defects per Unit Nanotube Length 1/*L*_a_ (1/μm)
P-SWCNTs	3.06	0.28	10.93	3.57
F-RT-3m	2.75	0.12	22.92	8.33
F-RT-4h	2.78	0.03	92.67	33.33
deF-RT-3m	3.09	0.15	20.60	6.67
deF-RT-4h	2.96	0.07	42.29	14.29

**Table 3:** Conductivity of each sample at 25 °C before and after the temperature rise.

Samples	Conductivity at 25 °C in Vacuum before Applying Heat (S cm^−1^)	Conductivity at 25 °C in Vacuum after Applying Heat (S cm^−1^)
P-SWCNTs	26.20 ± 3.22	22.66 ± 1.04
deF-RT-3m	8.92 ± 0.05	10.25 ± 0.29
deF-RT-4h	1.90 ± 0.10	1.71 ± 0.01

**Table 4:** Percentage of metallic nanotubes in each sample.

Samples	Integral Intensity		Percentage of Metallic SWCNTs
	*I*M_11_	*I*S_22_	
P-SWCNTs	4.84	34.19	12.40
deF-RT-3m	4.70	30.89	13.21
deF-RT-4h	0.97	12.87	7.00

## Data Availability

Not applicable.

## Supplementary Materials

The following supporting information can be downloaded at: https://www.mdpi.com/article/10.3390/nano13061086/s1, Figure S1: XPS (a) Si 2*p* and (b) O 1*s* narrow spectra of all samples; Figure S2: Nanotube length distributions of the (a) P-SWCNTs, (b) F-RT-3m, (c) F-RT-4h, (d) deF-RT-3m, and (e) deF-RT-4h.
