# Supplementary material for: Are Non-Six-Membered Ring Defects Formed in Single-Walled Carbon Nanotubes Treated by a Fluorination–Defluorination Process?
Source: Nanomaterials (Basel). 2023 Mar 17;13(6):1086. doi: 10.3390/nano13061086 (PMC10056250; doi:10.3390/nano13061086)
Supplement: Supplementary file 1 [file nanomaterials-13-01086-s001.zip › nanomaterials-2231431-supplementary.pdf]

Supplementary Materials

Are Non-Six-Membered Ring Defects Formed in Single-Walled Carbon Nanotubes Treated by a Fluorination–Defluorination Process?

Yoji Omoto[1], Hiromu Morita[1], Yoshinori Sato[2], Tetsuo Nishida[2], Kenichi Motomiya[1], Hirokazu Katsui[3], Takashi Goto[4,5], Yoshinori Sato[1,]*

[1] Graduate School of Environmental Studies, Tohoku University, Aoba 6-6-20, Aramaki, Aoba-ku, Sendai 980-8579, Japan

[2] STELLA CHEMIFA CORPORATION, 7-227, Kaisan-cho, Sakai-ku, Sakai, Osaka 595-0982, Japan

[3] Institute for Materials Research, Tohoku University, 2-1-1 Katahira, Aoba-ku, Sendai 980-8577, Japan

[4] Extreme Energy-Density Research Institute, Nagaoka University of Technology, 1603-1 Kamitomioka, Nagaoka, 940-2188, Japan

[5] New Industry Creation Hatchery Center (NICHe), Tohoku University, Aoba 6–6-10, Aramaki, Aoba-ku, Sendai 980-8579, Japan

[*]Corresponding author: Yoshinori Sato (yoshinori.sato.b5@tohoku.ac.jp)

Contents

1. XPS (a) Si 2*p* and (b) O 1*s* narrow spectra of all samples (Figure S1) p. 3

2. Nanotube length distributions of all samples (Figure S2) p. 4

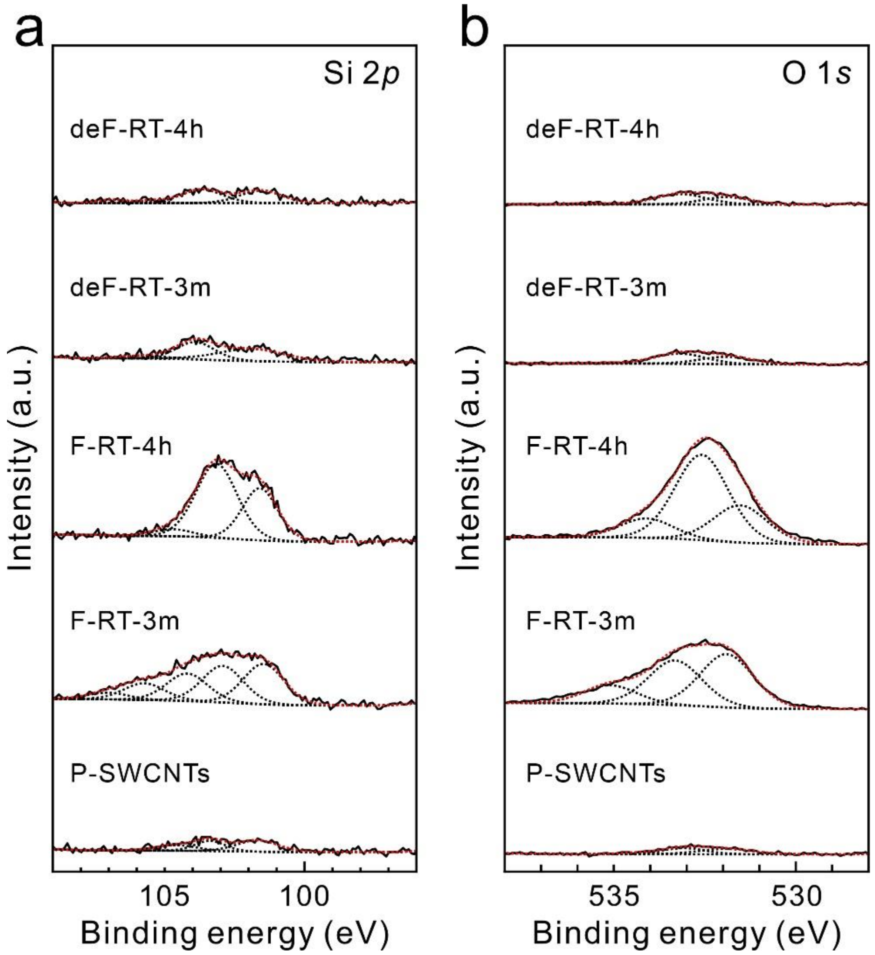

Figure S1. XPS (a) Si 2*p* and (b) O 1*s* narrow spectra of all samples. The black and red dotted lines represent deconvoluted peaks and fitted curves, respectively.

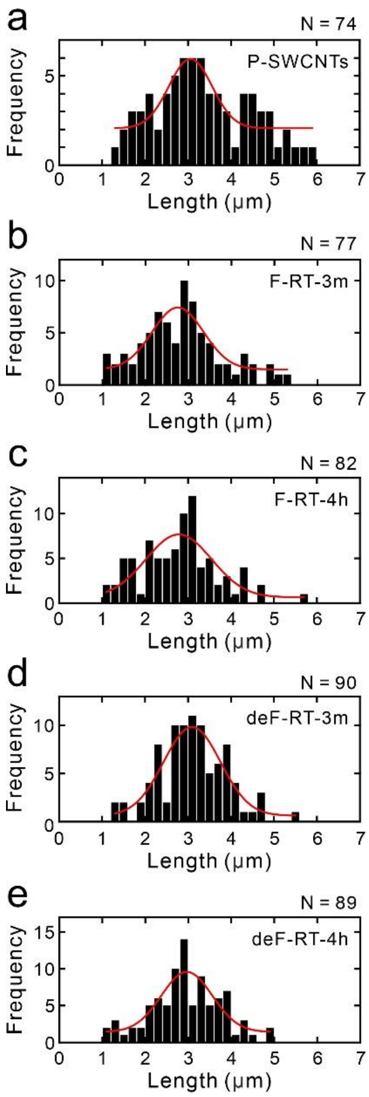

Figure S2. Nanotube length distributions of the (a) P-SWCNTs, (b) F-RT-3m, (c) F-RT-4h, (d) deF-RT-3m, and (e) deF-RT-4h.
